# Effect of wealth inequality on child and infant mortality in Togo

**DOI:** 10.1186/s12913-022-08912-4

**Published:** 2022-12-09

**Authors:** Essohanam Pelenguei, Mikémina Pilo

**Affiliations:** 1 Centre de Recherche en Economie Appliquée Et Management Des Organisations (CREAMO), University of Lomé, Lomé, Togo; 2 Laboratoire de Recherche en Sciences Economiques Et de Gestion (LaRSEG), University of Kara, Kara, Togo

**Keywords:** Infant and child mortality, Proportional hazard model, Wealth inequality, Togo, I14, D63, C14

## Abstract

At birth, individual has a health capital that depends on family, environmental and personal characteristics which depreciates over time requiring investment. It’s in this sense that this article aims to analyze the effect of wealth inequality on infant and child mortality in Togo. This effect is accessed by a semi-parametric proportional hazard duration model of Cox. According to the model estimation which is based on data from the Multiple Indicator Cluster Survey (MICS) carried out in Togo in 2017, the results obtained show in one hand that coming from a less wealthy household increases the risk of death for children. On other hand, the results show that the possession of a source of drinking water, the possession of health insurance by the mother, and the mastery of the use of new information technologies and communication reduce the risk of infant and child mortality. In view of these results, policies to reduce wealth inequalities could help reduce the risk of infant and child mortality in proportions ranging from 0.075 to 0.264.

## Introduction

The health of an individual depends on a set of family, environmental and personal characteristics including her personal or household wealth [1–3]. The relationship between wealth inequality and the health of population emerges in several studies in health economics and the demonstration of the causal link interest several researchers and economic and social development stakeholders [4–6]. Theoretical studies reveal three trends, which emerge from the analysis of the relationship between wealth inequality and health of populations.

The first trend, defended by authors such as [7] argues that inequalities positively affect the health of the population. For this group, taking into account inherited assets as wealth, person's sanitation and their health improve when inheritance inequalities increase. This trend bases its analysis on household wealth and shows that by taking into account the assets inherited by adults, individuals anticipate the repercussions that inequalities may have on health and thus improving health indicators in the long term.

The second trend argues that inequalities have no influence on an individual's health [8, 9]. This trend shows that the evolution of inequalities does not affect the demand for factors that determine health capital. Followers of this current claim that health indicators such as mortality and the behavior of individuals in relation to health care are hardly influenced by this trend by the fluctuations observed in the distribution of wealth or family income.

As for the third trend, it consists of pessimists about the effects of inequalities on the health of the population [10]. This group shows that the existence of wealth inequality equivalent to family income increases the mortality rate for both adults and children. Based on the theory of inequalities, they prove that the large dispersion of family income leads to a deterioration in the physical and mental health of these people. These pessimists establish the relationship that increasing wealth inequality, increases mortality, decreases the body's self-defense, causes chronic diseases, deteriorates blood pressure and also accentuates respiratory diseases.

Alongside these controversies, inequalities persist at all levels and are even growing. The top 10% in the world own 76% of total household wealth and dispose of 52% of total income for the same year (WIR, 2022). These observed inequalities are felt first between individuals and also between countries or regions of different given areas. Wealth inequality measured between adult individuals using market exchange rather than purchasing power parity reflects a higher level of inequality with the world's bottom 50% owning less than 1% of the total wealth and the world's top 10% who alone have almost 82% of the world's wealth. However, we note that sub-Saharan Africa, South and South-East Asia, Asia and Latin America have only 20 to 50% of average global wealth versus 50% to 100% for income. In Togo, the latest indicators show that in 2021, the top 1% of Togolese alone hold 12.2% of the national wealth against 13.9% held by 50% of the population belonging to the lower class [11].

With regard to the health of the population, even if considerable efforts are increasing day by day to achieve the second target of the third sustainable development goal with regard to the morbidity and mortality of children under 5 years of age, important disparities are still observed at the country level. Sub-Saharan Africa is the region where the mortality rate for children under 5 years is still the highest in the world, with an average of 74 children who die before celebrating their fifth birthday per 1000 live births, a delay of 20 years compared to the world average and 30 years compared to the rate recorded in West Asia and North Africa combined.

In view of the context presented above, this article seeks to answer the question: what is the effect of wealth inequality on infant and child mortality (ICM) in Togo? Through this question, we aim to analyze the effect of wealth inequality on infant and child mortality in Togo.

Starting from this objective and basing ourselves on the theory of inequalities, we formulate the hypothesis according to which the increase of wealth inequalities increases the infant and child mortality rate.

The added value of this research is twofold. First, to our knowledge, this is the first attempt to estimate the effect of wealth inequality on infant and child mortality in Togo. Unlike previous studies, we take monetary and non-monetary wealth for our analysis. The second level of added value is about the the model used. We integrate the duration separating the birth and the death of a child which allows us to identify in this study the law of probability of random variables by a distribution function which takes into account observable and truncated characteristics of each child at a given moment of their existence.

The rest of the article is structured as follows. Section 2 provides a brief literature review of the effect of wealth inequality on infant and child mortality. Section 3 presents through the analytical framework, the different channels through which wealth inequality affects child health. Section 4 is reserved for the presentation of the methodological framework and finally Sect. 5 presents then discusses the results obtained. The article ends with a conclusion.

## Brief literature review

The theory of social capital circumscribes the individual in the behavior he adopts. This theory shows that the individual behaves by being rational in his social choice [12]. It identifies household living standards, education, household size and structure as determinants of individual health outcomes. The theory of the reference model and social disorganization intervenes to describe a certain number of individual behaviors. An individual acts or behaves because they find it logical and socially acceptable [13]. The analyzes of these theoreticians thus appeal to the social disorganization which could explain the high frequency of births in irregular situations. Human behavior is actually an interaction between personality (individual characteristics), the influence of the family environment, that of the behavior of friends, and that of the social context in which the person lives [14]. Thus, the theory requires a fundamental study by deciding on a certain number of approaches such as the socio-economic approach with the focus on the standard of living “level of wealth”, the cultural approach and the demographic approach. These three theoretical approaches are complementary. The first explains better the transmission mechanisms of the effects of wealth inequality on the health of the population controlled by the other two approaches. The excess mortality of children who have not yet celebrated their first birthday is explained, according to the theory, by the lack of access to good food and basic social services (education, water, sanitation, etc.).

Empirically, several studies that have tried to establish the relationship between wealth inequality and the health of populations in developing countries have come to the conclusion that there is a negative relationship between wealth inequality and public health [15–17]. On the other hand, studies conducted by [18] show that in developing countries, changes in the level of wealth inequality do not in any way modify the health of populations. Research into the impact of income and wealth inequality on the health of an individual or population has been researched [19]. These studies are conducted on specific health outcomes and they show significant associations. These studies show that wealth inequality has a significant impact, among other things, on life expectancy [20], infant mortality [21], the Depression [22], mortality risk [23]. The different forms of inequality affect in different ways the health of individuals in general and that of mothers and children in particular [15, 17]. Maternal health is particularly associated with access to basic services such as water, sanitation, electricity, availability of housing, and access to health care.

Some analyzes identify a number of factors that interact on maternal mortality, such as the mother's education, health insurance coverage, health expenditure per person and the level of poverty [24]. Based on a bivariate and multivariate method, [25] come to the conclusion that the state of health of an individual depends on his level of wealth. For them, having a high level of wealth allows the individual to increase their health satisfaction, which abounds in their overall state of health.

## Conceptual framework of the influence of wealth inequality on ICM

**Fig. 1:**
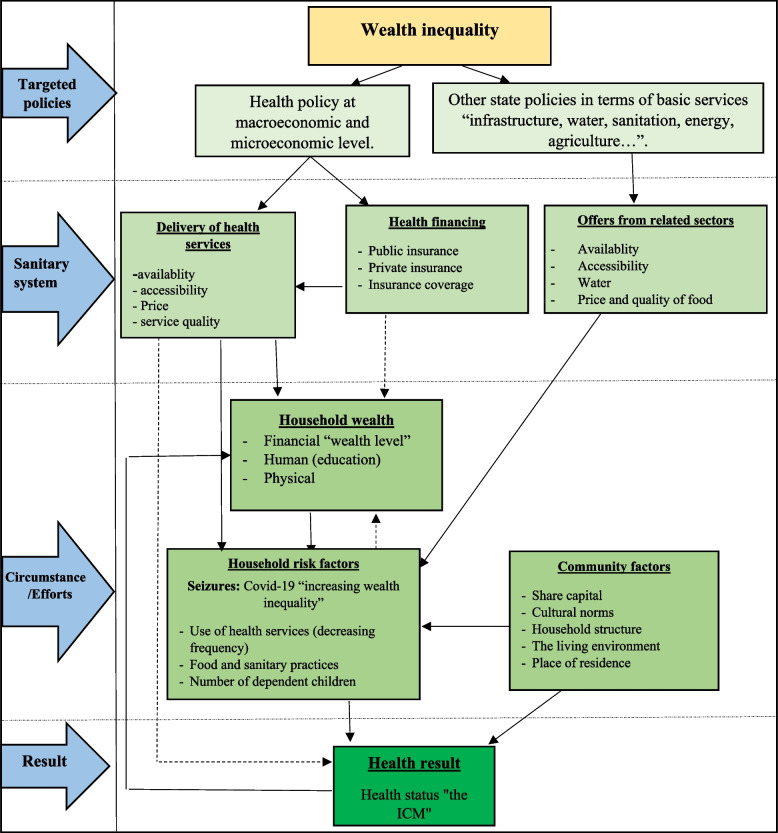
Analytical framework of the relationship between wealth inequality and mortality. Source: Authors, adapted from wagstaff, 2001

Within the framework of this research, we propose, through a conceptual framework, to analyze the transmission channels of the effects of inequalities of wealth on the health of an individual, taking in particular the mortality of children presented in Fig. 1. The existence of inequalities of wealth influence in certain specific conditions the mortality of individuals according to the theoretical developments [12, 13]. This influence begins with a gap in policies to be implemented in different localities within the same territory. Wealth inequality is accompanied by a health system and related actions to act on mortality. In the presence of these inequalities, among others, the provision of health services and health financing are solicited differently. The share of these services becomes a conflict that arises between the different classes resulting from the poor distribution of wealth. Rather, these services are supposed to increase the health capital of living individuals to keep them alive and extend their life expectancy. The conflict engendered by the increase in these inequalities increases household risk factors. These risks keep households in distress. The rise of new crises such as Covid-19 impose new rules of conduct on humanity, affecting the level of inequalities through the same channel. The health of the individual is therefore a result of social capital, cultural norms, household structure, living environment and place of residence [14]. These selected factors, which take into account the realities of African societies, are based on decisions on the use of health services according to a given frequency, food and health practices and also the number of dependent children, which is not negligible if one wants to study a phenomenon in a developing country like ours.

## Methodology

### Nature and source of data

#### Data sources

For our estimates, we use data from the Multiple Indicator Cluster Survey (MICS6) conducted in Togo in 2017 by the National Institute of Statistics and Economic and Demographic Studies (INSEED). This is a household survey containing a section of questions for household and children under five years. Among the 8,065 households found for the information-gathering sessions, 7,916 were surveyed in order to obtain information on the situation of children and women throughout the country. In these households, 5,030 children were under 5 years old and 4,942 of them has been successfully interviewed. A large number of variables were filled in as part of this survey. However, for the purposes of this article, only the variables presented in Table 1 have been retained. A large number of variables were filled in as part of this survey.

**Table 1:** Description of variables

Acronym	Description of Variables	Variable measures	Sign
**Dependent variables**			
**ICM**	Infant and child mortality	Child's age (0–5 years)	
		Age at death (0- 4 years)	
		State of life (0. Deceased 1. Alive)	
**IM**	Infant mortality	Child's age (0–1 year)	
		Age at death (0–11 months)	
		State of life (0. Deceased 1. Alive)	
**Variables of interest**			
**INEGR**	Wealth inequality	Poorest 10%; 2. D5 median; 3. Top 10%	** + **
INEGR*Residence	Wealth inequality and individual residence	Urban*poorest 10%; 2. Urban*D5 median; 3. Urban*Top 10%	** + **
**Control variables**			
**Sex**	The sex of Child	1. Boy; 2. Girl	** ± **
**Residence**	Place of residence	1. Urban; 2. Rural	**-**
**EducM**	Mother Education	1. None; 2. Primary; 3. Secondary and above	**-**
**Water**	Source of water that the household uses	1. Drinking water and 0 if not	**-**
**Insurance**	Possession of health insurance by the mother	0. No health insurance; 1. Has health insurance	**-**
**ICT**	The use of Information and communication technology by the mother	0. Does not use ICT; 1. Uses ICT	**-**
**Visit**	Number of prenatal visits made by a woman before giving birth	Continuous variable	**-**
**Cut**	Household size	Continuous variable	** + **
**Childbirth**	Previous delivery	1. Has ever given birth to a child and 0 If no	**-**
**Religion**	Religion attended by child’s mother	1. Christian; 2. Muslim; 3. Animist	** ± **
**Twins**	Child Birth Type	1.Twin; 2. alone	** + **

Source: Authors based on data from MICS, 2017

#### Description of variables justification of their choice

The variables retained for our article are chosen on the basis of literature and theory. These variables are recorded in Table 1 where we also mention the expected sign of each of the variables.

### Model specification

The estimation of health indicators taking into account wealth disparities and a set of control variables has been theorized by some authors through specific models [18, 26, 27].

1 $${IS}_{i,t}= {\alpha }_{0}+{\beta INEG}_{i,t}+{\theta y}_{i,t}+{\gamma X}_{i,t}+{\varepsilon }_{i,t}$$

${IS}_{i,t}$ represents the health indicators of an individual i at time t “life expectancy at birth, infant mortality, infant and child mortality rate”; $\beta$ is the coefficient associated with wealth inequality; $\theta$ the coefficient which measures the influence of the individual characteristics on his health results; $X$ the set of covariates identified by the literature that can determine the state of health of a population and $\varepsilon$ the random error term that takes into account all the other variables that the estimate does not then capture.

Through this theoretical model, we propose by adapting, an empirical model which allows us to estimate our various coefficients. The use of such a model requires taking into account a set of parameters in accordance with the semi-parametric model of Cox. This model is the most used to capture the risk of occurrence of an event in the life of an individual. We find as fundamental hypothesis of this method the risk of death of a child which is only a function of his age. In addition to this methodological approach, [28] develops a proportional hazard model that may be invalid in case the proportionality of risk assumption is not verified. We are interested in the semi-parametric model “proportional hazards model” which is based on flexibility and ease of use. This advantage offered by this model allows us to take as an unexpected event in the lives of children their mortality before the age of 5 and at the same time we highlight the effects of the poor distribution of wealth on the results.

We also introduce certain characteristics specific as the structure of the household and its size, social cohesion, the number of visits to prenatal care for the child victim and the possession of health insurance.

The estimate of the instantaneous risk conditional on the various covariates is expressed as follows:

2 $$\delta \left(\frac{t}{{X}_{i1}},\dots .. , {X}_{in}\right)={\delta }_{0}\left(t\right)\mathrm{exp}\left({\beta }_{1}{X}_{1}+\dots +{\beta }_{i}{X}_{i}\right)$$

3 $${=\delta }_{0}\left(t\right)\mathrm{exp}({\beta }_{i}{X}_{i})$$

With ${\delta }_{0}\left(t\right)$ an unknown function and function of time representing the basic risk; $\beta =\left({\beta }_{1},\dots \dots ., {\beta }_{i}\right)\epsilon R$ is a set of unknown parameters of dimension i independent of time ${\varvec{t}}$. It represents the effect of the covariates on the instantaneous risk and $\mathrm{exp}({\beta }_{1}{X}_{1}+\dots +{\beta }_{i}{X}_{i}$ represents the relative risk. It is then question in this study, to empirically determine the effects of the explanatory factors on the variation of the risk through the estimation of the parameters.${\beta }_{1},\dots \dots ., {\beta }_{i}$

#### Kaplan Meier curve estimation method

Through this method, we estimate the distribution of the survival function, i.e. the distribution over time of the probability of not having experienced infant and child mortality. To do this, we take into account indicators such as:

- ${t}_{i}$ an instant during which there is the observation of at least one death
- ${d}_{i} and {c}_{i}$ the numbers of individuals “children under five years old” who, respectively, know the event “death” and leave observation (right censoring) at ${t}_{i}$
- ${N}_{i}$ the population “children under five on the one hand and under one on the other hand” submitted to the risk at time

We therefore deduce two empirical models that correspond respectively to the estimation of the effects on infant and child mortality and infant mortality:

Model 1: infant and child mortality

4 $${ICM}_{i}={\beta }_{0}+{\beta }_{1}Inegr+{\beta }_{2}Sex+{\beta }_{3}Residence+{\beta }_{4}Educm+ {\beta }_{5}water+{\beta }_{6}insurance+{\beta }_{7}TIC+{\beta }_{8}Visite+{{\beta }_{9}size+{\beta }_{10}Accouchement+\mu }_{i}$$

Model 2: Infant mortality

5 $${MI}_{i}={\beta }_{0}+{\beta }_{1}Inegr+{\beta }_{2}Sex+{\beta }_{3}Residence+{\beta }_{4}Educm+ {\beta }_{5}water+{\beta }_{6}insurance+{\beta }_{7}TIC+{\beta }_{8}Visite+{{\beta }_{9}size+{\beta }_{10}Childbirth+\mu }_{i}$$

### Risk proportionality test

**Fig. 2:**
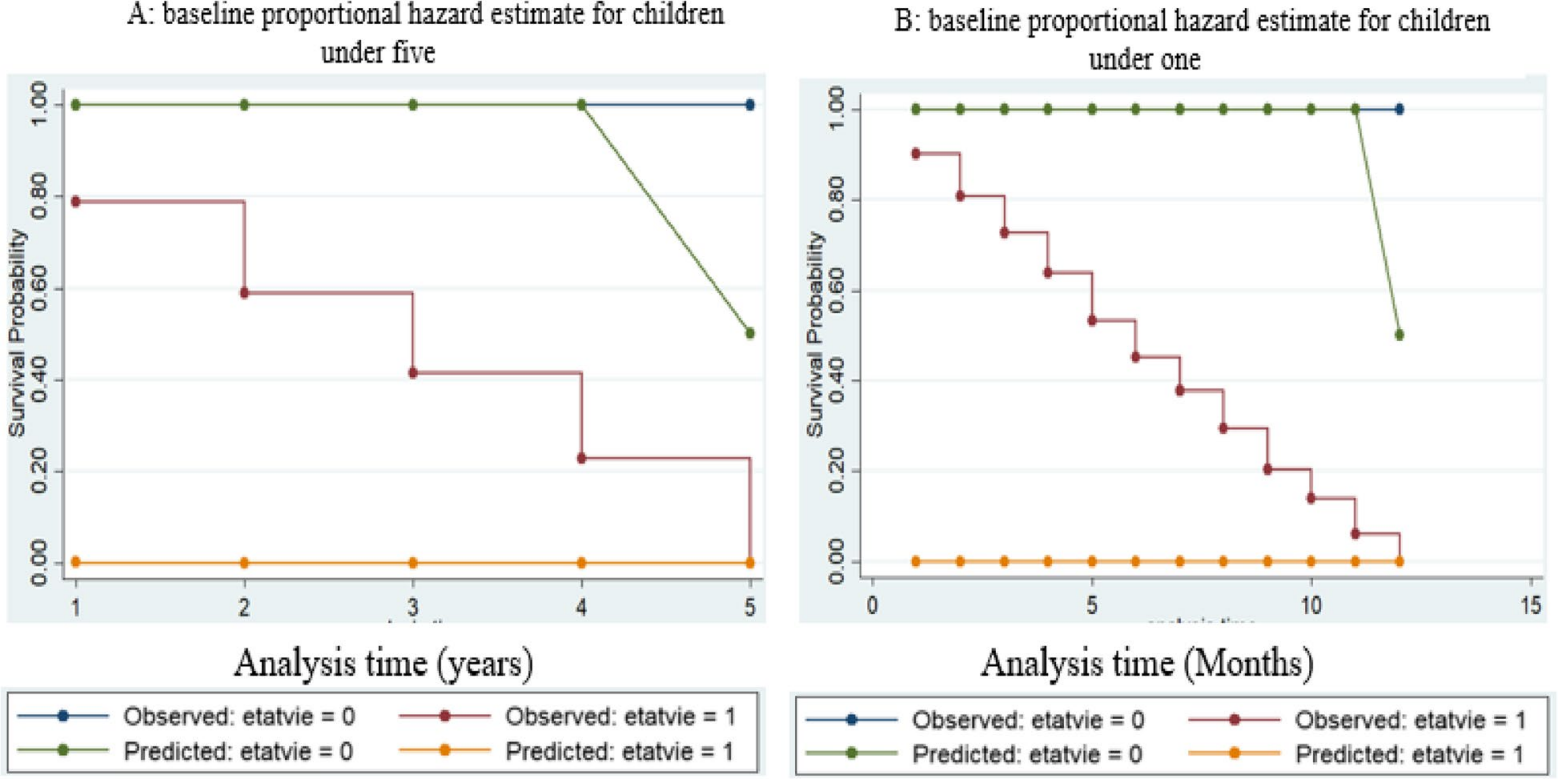
Baseline proportional hazard estimate for children under five and one year old. Source: Authors based on data from MICS, 2017

We first test the risk so that each child at a specific moment in the analysis can have the same chances of survival or run the same risk of death. This is one of the fundamental assumptions that the Cox model requires before it is used. The consistency of the difference between the predicted curves and those observed throughout the analysis period is then checked; four years for the figure on the left and 11 months for the figure on the right. Figure 2 indicates through the parallelism of the two predicted curves that there is a proportionality of the risks over the four years and eleven months following the birth of these children.

## Results and discussion

This section first presents the statistical analysis, then the results of the prediction tests of the model and finally the econometric results.

### Analysis and interpretation of statistical results

#### Statistical analyzes

Table 2 presents the overall socio-demographic characteristics of the households included in the analysis. The chi-square test (Coron, 2020) significant at the 1% threshold confirm the association between ICM and another variable introduce in the model. The number of children who were the subject of the analysis is 11,731. These children are both “boys or girls” and come either from rural or urban areas. Our statistical analysis shows that the health outcomes of those children who are under age 5 years depend on the education of their parents. The data show with regard to infant mortality that, it’s boys who have the highest quotient “44 children out of 1000 live births die between their birth and their first birthday” against a quotient of 39 for girls. This quotient is lower when the level of education increases “25 deaths per 1000 live births for a secondary level and more against 48 for those with no level. Happiness statistics show that men are more unhappy than women (27.5% versus 18.8% respectively). Concerning child mortality, no difference is revealed in terms of the quotient recorded for girls as for boys. The number of children aged between 1 and 4 years per 1000 surviving children is higher when with uneducated parents. This quotient is 42 against 13 surviving children for children whose parents have at least secondary education. Observation of the data recorded in Table 2 also shows that the infant and child mortality rate is higher in rural areas (82) than in urban areas (53 deaths per 1000 live births. concerning the wealth inequality variable, our results are in percentage of survival. The statistics also show concerning wealth inequality that, in poorest households, respectively 89.94% and 76.39% of children survive beyond their first and fifth anniversary. However, these rates rise when it comes to the wealthiest. These are respectively 95.21% and 93.84% children who manage to celebrate their first and fifth anniversary.

**Table 2:** Descriptive statistics of the variables

	Infant mortality quotient	Children mortality quotient	Infant and Children mortality quotient
**Chi-squared**	Prob > Chi2 (0,000)		Prob > Chi2 (0,000)
	*P*value: 103, 260		*P*value: 220, 60
**Child Sex**			
Boy	44	31	74
Girl	39	31	69
**residence**			
Urban	35	19	53
Rural	46	38	82
**Mother education**			
None	48	42	48
Primary	47	30	47
Secondary and above	25	13	25
**Wealth inequality**	(%)	(%)	(%)
Poorest 10%	89.94	70,11	76.39
D5 Median	91.43	80,23	82.86
Top 10%	95.21	90,15	93.84

Source: Authors based on data from MICS, 2017

#### Kaplan–Meier estimation of survival probability

**Fig. 3:**
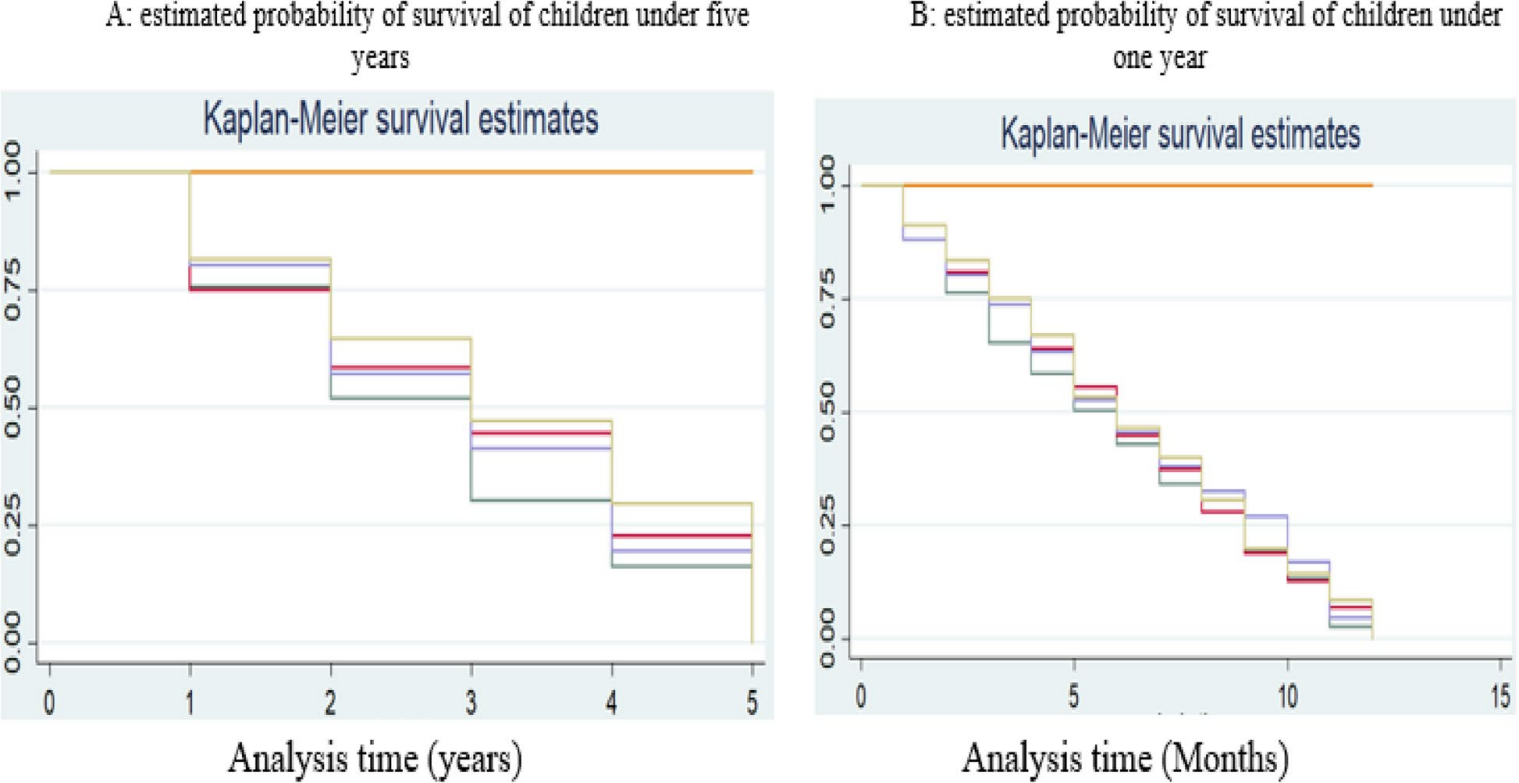
Estimated probability of survival of children under 5 years and one year. Source: Authors based on data from MICS, 2017

We present through Fig. 3, the evolution of the probability of survival of children during the first eleven months and the fourth years following their delivery. The stairs in this figure represent the probability of survival during the different periods of death “four years or eleven months of observation”. Each height of a stair describes the risk of a child to die at some point in the analysis. The observations are made per year for the figure on the left (Fig. 3A) and per month for the figure on the right (Fig. 3B). This figure shows us, taking into account the speed of occurrence of deaths, the probability of survival of children.

Concerning the probability of survival of children under 5 years, the evolution is more or less regular. Over the four years following the birth of the children, they have at each birthday almost the same probability of survival. This probability follows a steady trend during the first four months after childbirth before registering a drop in the fifth month. This month represents the modal class of children under the age of one who record a rapid but not regular fall. Beyond this fifth month, the function of survival continues its regular fall until the 11th month.

### Analysis and interpretation of econometric results

We present the statistical results from our estimates in this section.

#### Effects of wealth inequality on ICM

The econometric estimates made it possible to highlight, among the variables that were integrated into our model, the factors that explain infant and child mortality (Table 3). These show that the coefficients are mostly significant at the 1%, 5% and 10% threshold. Differences in the wealth held by households affect the death of children under five. We based our interpretation on the distribution of wealth by using the deciles. this distribution goes from the poorest 10% to the richest 10% through the median. Our results suggest that when a household is in the median wealth decile, the risk that a child in that household die before reaching their fifth birthday decreases by 4.29%. it is the same with children living in 10% of wealthiest household. These children’s chances of surviving are 14.41 higher than those of the poorest 10%. This chance is 33.41% for those under one year old. These results are consistent with those of [7, 29]. This can be explained by the fact that wealth enters into the acquisition of health capital which improves with the purchase of adequate health services. Recently in 2019, nearly half of the Togolese population lived below the poverty line (i.e., 45.5% of Togolese) (INSEED, 2020). Those living on less than $1.9 a day will not be able to truly acquire the health capital necessary for their children, unlike those who live with a much higher level of wealth. The health capital that deteriorates over time with age will quickly depreciate and cause poor health in children and consequently death before they can celebrate their fifth birthday.

**Table 3:** Cox Survival Model Proportional Hazard Regression

Variables	Model 1 (relative risks)	Model 2 (relative risks)
Wealth inequality (Poorest 10%)		
D5 Median	0,9571^b^	0.9266^b^
Top 10%	0.8759^c^	0.6659^c^
Sex (Girl)		
Boy	0.858^c^	0.9213^c^
Residence (Urban)		
Rural	1.0111	0.8549^c^
Mother education (None)		
Primary	0.964^b^	0.9610^c^
Secondary and above	0.904^b^	0.9147^c^
Drinking water (not potable)		
Clear water	1,157	0.9571^c^
Insurance (Without insurance)		
Has a health insurance	0.650^c^	0.9196^b^
Household size (less than 4)		
Four and more	1,0436^c^	1,024
Delivery (Never)		
At least one Childbirth	0.9902^c^	1.018
ICT (not using)		
Using ICT	0.993^a^	0.964^b^
Prenatal visit (under 4 visits)		
More than 4 visits	0.900^c^	0.8830^c^

Model 1 is for children (under 5 years) and model 2 for children under 1 year

^a, b, c^denote 1%, 5% & 10% level of significance

Source: Authors based on data from MICS, 2017

The analysis of our results also shows that the risk for a boy to die during the five years following childbirth decreases by 14.2 than that of a girl.

The level of education contributes to infant and child health. According to the results of the regression, the risk of infant and child mortality is lower when the mother of the child has a primary or secondary level of education and more compared to those who have no level of education. That said, children born to mothers with at least secondary level of education are 9.6% less likely to die than those born to mothers with no schooling at all. This result is similar to some works by [30]. In our context, the springs find foundation in the fact that the education of an individual has an advantage which is much more economic. Education leads to greater awareness of health rules,

Among these economic benefits of education is insurance coverage. Our results show that the risk of infant and child mortality decreases with the possession of health insurance. A child born to a woman who has health insurance has 35% less likely to die than those colleagues whose mothers have no health insurance. This result is consistent with those of [31] in Rwanda.

The results obtained through the estimation of the effect of water suggest that using drinking water in the concession does not reduce the risk of infant and child mortality. This result is counterintuitive and does not corroborate with the results found by [31] for whom water, sanitation and even hand washing reduce the risk of death in Ethiopia. This is a result that may be due to the non-representativeness of the individuals in our analysis who use spring water in both urban and rural areas (24.6%). In contrast to the effect of water on the health of children under five, the Model 2 estimate proves that using clean water in the home for basic needs helps reduce the risk of mortality children under one year old. Our analysis shows that the chances of a child surviving beyond eleven months postpartum are 0.0429 higher than those using water which is not safe to drink [31, 32]. With the advancement of the rules of medicine, the seventh month of the link between the type of water and the seventh month of the child explains this importance insofar as this child will have to start drinking water, which affects his organization directly.

Depending on the size of the household, the risk of infant and child mortality increases proportionally. We also find that a child born in a household where there are more than four children in charge has 4,36% more likely to die before celebrating his fifth birthday than those with a maximum of four children. This is explained by the fact that the importance that should be given to each child decreases as they become numerous; which is consistent with the results of the work of [33, 34].

The changing world of technology is also helping to improve child health. Our study shows that new information and communication technologies such as the use of smartphones or tablets by one of the parents improves the health of the latter. When a parent has a mobile phone or tablet, children have 0.7% more likely to be able to celebrate their fifth birthday more than those whose parents do not have such a digital tool. These tools allow appointments to be made, for example for vaccinations and reminders, as we have seen with the booster doses against the Covid-19 pandemic in Togo and everywhere else.

Our results also prove that having already procreated and made a prenatal visit reduces the risk of infant and child mortality. The risk that a child who is less than five years old dies is 0.021 lower when the mother has previously had at least one child. This result is nothing more than the experience that mothers acquire before, during and after previous deliveries to better take care of future births. This is felt much more through the domestic assistance of mothers to their daughters ("young mothers") which are in need at the first Birth. From the second childbirth, the mother-in-law or the mother of the one who gave birth may no longer be present because of the experience already acquired by the latter. Also having followed the four prenatal visits of the WHO during pregnancy has advantages for the child under five years of age. These has 10% less likely to not celebrate their fifth birthday alive when his mother has made more than four visits than their counterpart whose mothers have made less than four visits. This result is consistent with those of Ahmed and al., 2010 in developing countries.

Place of residence being one of the controversial factors in the two models, we determine through a third model the effect that an interaction of wealth inequality and place of residence may have on infant and child mortality.

#### Effects of the interaction “wealth inequality and place of residence” on ICM

The estimation of the interaction model is presented in Table 4. This part consists of highlighting the effects of wealth inequality while taking into account the place of residence of people. We add in this section an aspect of African society particularly Togolese, that of the treatment accorded to twin children. Our estimate shows that wealth inequality has a different influence on infant and child mortality depending on whether one living in an urban or rural environment. The results obtained following this estimation prove that when a child is born in median 10% wealthy households in urban areas, the probability to die before celebrating their fifth birthday decreases by 7.3 percentage points compared to the poorest in rural areas. This probability is lower for children living in top 10% wealthiest household in urban areas. For children in this category, the probability to die before celebrate their fifth anniversary decreases by 8,1 percentage points than the poorest 10% in rural areas. These results seem similar to those of [35] and contradict the findings of [36]. They find no evidence of a statistically significant difference in the risk of infant and child mortality between households in the poorest and poorest wealth quintiles in urban areas and their rural counterparts in Sub-Saharan Africa. Our result can be explained in this context by the fact that in urban areas, there’s easy access to the medical technical platform necessary for emergency intervention in the event of a child's illness, easy mobility due to available infrastructure, reduced proximity to health centers which all contribute to reducing the risk of infant and child mortality. Besides this result, the estimation of this third model gives credence to the two previous ones with regard to the control variables used in the analysis. Among other things, it emerges from this estimate that the probability that a child dies before celebrating its fifth birthday decreases with the level of education of one of its parents. The probability for a child born to a mother with primary education to die before 5 years decrease by 3.9 percentage points than those with mothers not educated. The education of the mother makes it possible to better control the rules of hygiene, to better apply them, to follow the indications of the prescriber of drugs for a better health of the child. Similarly, factors such as a parent's health insurance and prenatal visits reduce the risk of infant and child mortality. These factors contribute to reduce the probability of infant and child mortality respectively by 30,86 percentage points and 1.87 percentage points more than those who do not have health insurance and those who make less than four prenatal visits during their pregnancy. The prenatal visits allow the mother to collect a certain number of essential essentials for the survival of the child as well as for her own health. This estimate also shows that children whose parents are Muslims or animists run less risk of dying during the 4 years following childbirth. The probability for a Muslim child to die before celebrating his fifth birthday is 15.79 percentage points lower than for Christians. Similarly, practicing a traditional religion or being an animist seems to reduce the risk of infant and child mortality in Togo by 21.1 percentage points [37, 38]. The cultural attachment, the bad interpretation of the biblical writing by the Christians can explain the increase of the risk these last make run with their children.

**Table 4:** Estimate of the mixed effect

	Marginal effects	P > Z	[95%Conf, Range]	
Wealth inequality^a^ Residence (Poorest 10%^a^Urban)				
D5 Median	-0.0733^b^	0.001	-0.115	-0.030
Top 10%	-0.081^a^	0.000	-0.219	-0.125
Sex (Girl)				
Boy	-0.237^c^	0.000	-0.104	-0.055
Mother education (None)				
Primary	-0.039^a^	0.000	0.084	0.144
Secondary and above	-0.065^a^	0.000	0.357	0.461
Type of delivery (single)				
Twins	0.1407^a^	0.497	-0.059	0.122
Insurance (Without insurance)				
Has a health insurance	-0.3086^c^	0.038	-0.134	-0.004
Prenatal visit (under 4 visits)				
More than 4 visits	-0.0147^c^	0.000	-0.154	-0.092
Household size (less than 4)				
Four and more	0.03848^c^	0.000	0.022	0.077
Religion (Christians)				
Muslim	-0.1579^c^	0.000	-0.151	-0.065
Traditional/animist	-0.2236^c^	0.000	-0.247	-0.178

^a, b, c^denote 1%, 5% & 10% level of significance

Source: Authors based on data from MICS, 2017

These aforementioned factors are for the most part of the order of humans who can, because of their choices, ask for less or more to contribute to the increase in the health capital of the child. Genetic factors that escape humans cannot be captured in the same way. So, we take into account the fact of being born alone or twins. Our result shows that being born twins increases the risk of infant and child mortality compared to children born alone. A child who is born twin has 14.07 percentage points more like than their counterparts who are born alone to die before their fifth birthday. This result seems to agree with that of [39]. Having twins requires a lot more effort to care for them, a lot more attention to them, and even minimal neglect is fatal for these children. In African traditions particularly in Togo, this can also be explained by the fact that the death of one of the twins leads to that of the other provided that he does not see him or is aware of it.

## Conclusion

This article is devoted to the analysis of the effect of wealth inequality on children's health, taking infant and child mortality as the target result, based on data from the MICS survey conducted in Togo in 2017. We used the semi-parametric model of Cox to achieve the objective pursued. Following our estimates, our results mainly show that when a household is in the middle wealth quintile, the risk that a child in this household die before celebrating their fifth birthday decreases. It should also be noted from our results that a child born to a mother who has completed secondary school or more less likely to die than those born to mothers with no schooling at all. In view of our results, a better distribution of wealth is an option to be favored to significantly reduce infant and child mortality in Togo. Policies aimed at improving children's health must also take into account the illiteracy of parents because they are already adults. Moreover, encouraging a moderate household size also remains a relevant strategy for reducing infant mortality.

## Data Availability

The datasets generated and analyzed during the current study are available on the demographic and health survey program page https://dhsprogram.com/data/available-datasets.cfm. Nevertheless, the download is subject to the study of a research project which justifies the need for this data.

## Abbreviations

ICM: Infant and Child Mortality
ICT: Information and Communications technology
IM: Infant Mortality
INSEED: National Institute of Statistics and Economic and Demographic Studies
MICS: Multiple Indicator Cluster Survey
UNICEF: United Nations of International Children's Emergency Fund
WHO: World Health Organization
WIR: World Inequality Report
